# Effect of the prior distribution of SNP effects on the estimation of total breeding value

**DOI:** 10.1186/1753-6561-6-S2-S6

**Published:** 2012-05-21

**Authors:** Javad Nadaf, Valentina Riggio, Tun-Ping Yu, Ricardo Pong-Wong

**Affiliations:** 1 DNA Landmarks Inc. St-Jean-Sur-Richelieu, J3B 6X3 Quebec, Canada; 2 The Roslin Institute and the R(D)SVS, The University of Edinburgh, Easter Bush, Midlothian, EH25 9RG, Scotland, UK

## Abstract

**Background:**

Five main methods, commonly applied in genomic selection, were used to estimate the GEBV on the 15^th ^QTLMAS workshop dataset: GBLUP, LASSO, Bayes A and two Bayes B type of methods (BBn and BBt). GBLUP is a mixed model approach where GEBV are obtained using a relationship matrix calculated from the SNP genotypes. The remaining methods are regression-based approaches where the SNP effects are first estimated and, then GEBV are calculated given the individuals' genotypes.

**Methods:**

The differences between the regression-based methods are in their prior distributions for the SNP effects. The prior distribution for LASSO is a Laplace distribution, for Bayes A is a scaled Student-t distribution, and the Bayes B type methods have a Spike and Slab prior where only a proportion (π) of SNP has an effect, following a given distribution. In this study, two different distributions were considered for the Bayes B type methods: (i) normal and (ii) scaled Student-t. They are referred here as the BBn and BBt methods, respectively. These prior distributions are defined by one or more parameters controlling their scale/rate (λ), shape (df) or proportion of SNP with effect (π). LASSO requires one (λ); two for Bayes A (λ, df) and Bayes Bn (λ, π); and three for Bayes Bt (λ, df, π). In this study, all parameters were estimated from the data. An extra scenario for Bayes A and BBt was included where df was not estimated but fixed to 4 (suffixed _4df). The implementation of GBLUP was done using ASREML, the heritability was also estimated from the data. All other methods were implemented using a MCMC approach.

**Results:**

All Bayes A and B methods showed accuracy (correlation between True and Estimated BV) as high as 0.94 except for BA_4df (r = 0.91). Compared to the traditional BLUP using pedigree information, these methods improved the accuracy between 50 and 55%. GBLUP and LASSO were less accurate (0.81 and 0.85 respectively) and the improvements were 34 and 40% compared to BLUP.

**Conclusions:**

Results of all methods were consistent and the accuracies for GEBV ranged between 0.81 and 0.94. When all parameters were estimated the results were similar for the Bayes A and Bayes B methods. Results showed that Bayes A was more sensitive to the changes in the shape parameter, and the parameter changes led to change in the accuracy of GEBV. However BBt was more robust to the change in this parameter. This may be explained by the fact that BBt estimates one extra parameter and it can buffer against a non-proper shape parameter.

## Background

With the availability of cost-effective SNP chips, genomic selection is currently been applied in agricultural species, using different statistical approaches. SNP information can be used to estimate breeding values by using (i) a mixed model or (ii) a regression-based approach. For the first approach, the SNP genotypes are used to estimate the relationship matrix among individuals, which later is used in a BLUP analysis (GBLUP) to estimate genomic breeding values (GEBV). For the second approach, the effect of individual SNPs can be estimated using multiple regression, and then GEBV for each individual are calculated as the sum of the SNP effects given their genotypes. Estimated SNP effects may also be of value to be used as a criterion for QTL mapping. Examples of methods based on this approach include Bayes A, Bayes B [1] and LASSO [2]. The main difference between these methods is their assumption on the prior distribution of the SNP effects. These distributions are defined by one or more parameters regulating their shapes and scales/rates and can be assumed known or, alternatively they can be estimated from the data itself.

The aim of this study was to compare the GEBV using different methods of genomic selection on the common dataset from the 2011 QTLMAS workshop. For the different methods, the values for the parameters regulating the prior distribution of the SNP effect were also estimated from the data.

## Methods

### Dataset

The dataset used here was the one provided by the organisers of the 15^th ^QTLMAS workshop. It consisted of 3220 individuals, all were genotyped for about 10000 SNP on five chromosomes of equal length (1 Morgan each). The pedigree included 20 half-sib and 200 full-sib families, each including 15 offspring. Eight QTLs were simulated to affect the quantitative trait, with the largest one on chromosome 1, 2 linked QTLs on chromosomes 2 and 3, 1 imprinting one on chromosome 4, and 2 epistatic QTLs on chromosome 5. Heritability of the trait was 0.3 and two third (n = 2000) of the offspring were phenotyped.

### Genomic evaluation

#### Mixed model based method: GBLUP

For the GBLUP approach, breeding values are assumed to be a random effect and included in a mixed model as the following:

y=μ+Zg+e

where **g **is the vector of random total genetic effect assumed to be normally distributed as N(**0**,**G**σ^2^_g_), with **G **being the realised relationship matrix calculated from SNP information [3], and σ^2^_g _the variance of **g**. The GBLUP was implemented using ASREML [4], in a two-step approach where σ^2^_g _was first estimated from the data and later used to calculate the GEBV.

#### Regression-based methods

The model for all these methods is the following:

$$y=\mu +\overset{n}{\underset{i}{\sum }}z_{i}\beta_{i}+e$$

where, n is total number of SNP, **z**_i _is the vector of genotypes at SNP i; β_i _indicates the allelic substitution effect for SNP *i*.

The difference between the regression-based methods lays on the assumption of the prior distributions for the SNP effects. For instance, the prior distribution for LASSO [2] is a Laplace distribution, for Bayes A [1] is a scaled Student-t distribution, and the Bayes B type methods have a Spike and Slab prior where a proportion (π) of SNP has an a non zero effect on the trait, and the remaining (1-π) SNP having no effect on the trait. For the SNP affecting the traits two different distributions were considered: (i) normal and (ii) scaled Student-t. Here, they are referred as the BBn and BBt methods, respectively. The BBn method has also been referred as Bayes C.

These prior distributions for SNP effects are described by one or more parameters which includes scale/rate (λ), shape (df) and/or proportion of SNP with effect (π). The prior distribution assumed with LASSO is defined by one parameter (λ); two for Bayes A (λ, df) and Bayes Bn (λ, π); and three for Bayes Bt (λ, df, π). In this study, all parameters were estimated from the data (suffixed _edf). An extra scenario for Bayes A and Bayes Bt was included where df was not estimated but fixed to 4 (suffixed _4df). The models were implemented under a Bayesian framework using Gibbs sampling. Bounded flat priors (between 0.5 and 9) were used for the estimation of df. The parameters π and σ^2^_SNP _were estimated from data using flat priors. For each analysis, a MCMC chain was run and the first 10000 cycles were discarded as burn-in period. Following this, 10000 realisations were collected, each separated by 20 cycles between consecutive realisations (i.e. length of chain = 210,000 cycles). The posterior mean was used as the estimate for each parameter of interest. For the case of Bayes A and B where df was estimated, the chain was 5 times longer.

#### Variance explained by genomic information

For this estimation, an approximation based on the infinitesimal model theory was used [5]:

$$\text{Var}(\text{EBV})=r^{2}\sigma^{2}{}_{\text{g}}$$

$$\text{PEV}=(\text{1}-r^{\text{2}})\sigma^{\text{2}}{}_{\text{g}}$$

$$\sigma^{2}{}_{\text{g}}=\text{Var}(\text{EBV})+\text{PEV}$$

where PEV is the Prediction Error Variance, and *r *is the accuracy of estimates. The explained additive genetic variance (σ^2^_g _) was obtained using the above equations, for regression-based methods and the corresponding heritability was reported.

### QTL mapping analysis

Additionally to the GEBV, the regression-based methods also estimate the effects for each individual SNP used in the analysis. We compared these results with association and linkage analysis results, in order to assess its potential use as a criterion for QTL mapping.

#### Linkage analysis

Half-sib analysis (HS-QTL) was performed as described by Haley et al. [6], and implemented in the GridQTL [7]. The analysis was based on studying the segregation of the paternal allele.

#### Association analysis

Association analysis was performed using the GRAMMAR approach [8], which comprises two steps. First, phenotypes were adjusted for the polygenic effects and second, residuals were fitted against each SNP using additive model as implemented in GenABEL [9].

## Results and discussion

### Genomic evaluation

**Figure 1:**
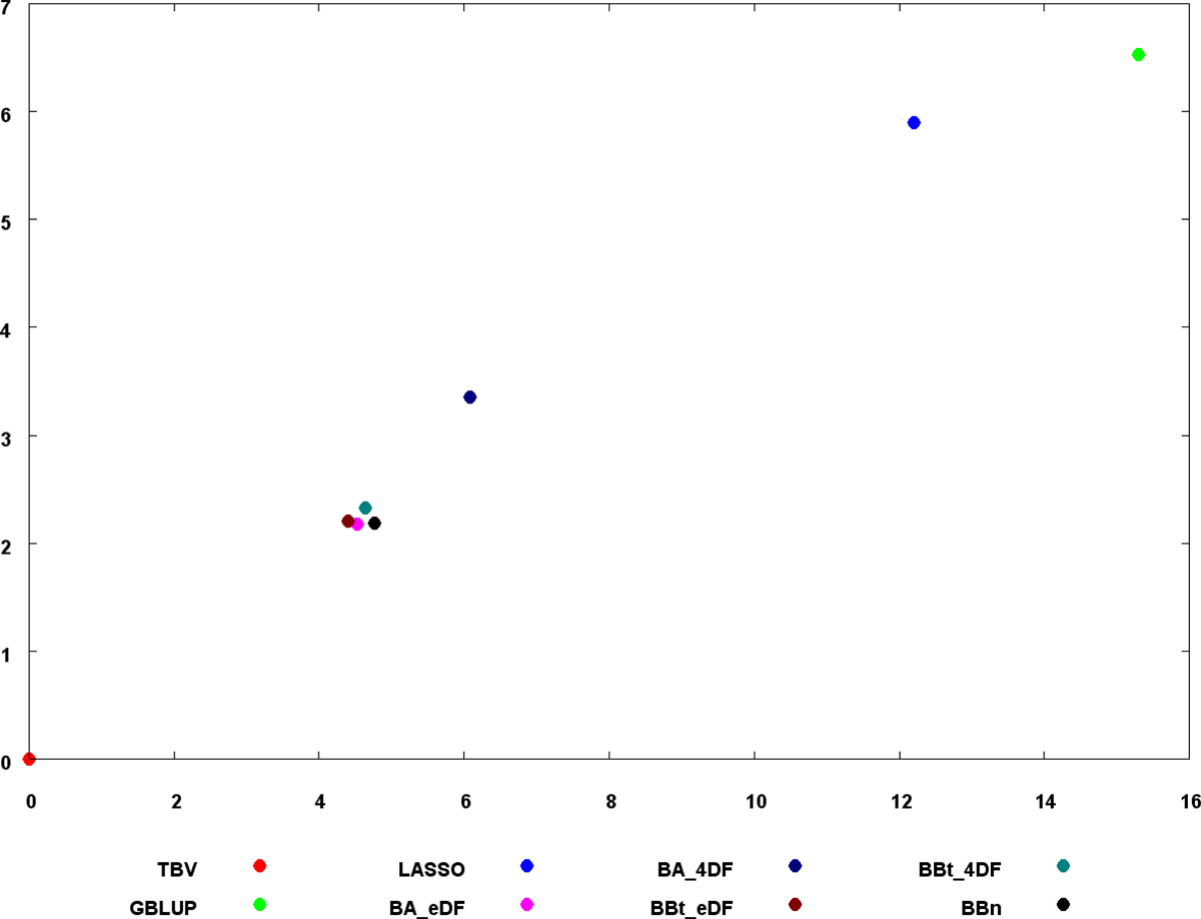
**Principal component analysis on GEBV for the different Genomic selection methods**. The values for the two largest principal components were rebased so the true breeding value fall at the origin.

Generally speaking, the GEBV with the different methods were consistent among themselves with the correlation between GEBV ranging from 0.898 to 1 (see scatter plot in Additional file 1). To further show the relative similarity between methods a principal component analysis was performed on the GEBV for all methods and results are reported in Figure 1. When the parameters of the prior distribution were estimated from the data, Bayes A and Bayes B type yielded the same results (see results for BA_edf and BBt_edf). The results for BBn were very similar to BBt_4df (*r*=0.999). LASSO and GBLUP had similar results (*r*=0.991) and slightly less correlated with the other methods.

The accuracy of the methods, expressed as correlation between TBV and EBV, are shown in Table 1. The Table also shows the improvement of the accuracy compared to the traditional BLUP, and heritability estimated by each method. Bayes A and Bayes B type methods showed the highest accuracy (*r*≈0.94) with BA_4df having a slightly lower accuracy (r = 0.91) representing an improvement between 50 and 55% compared to traditional BLUP. The accuracy of GBLUP and LASSO were 0.81 and 0.85, an improvement over BLUP of 34 and 40%, respectively. Heritability (simulated h^2 ^=0.30) estimated by all methods were very similar (~0.29), except for GBLUP (h^2^=0.27).

**Table 1:** Accuracies and heritabilities estimated by all methods.

Methods	*r*	Improvement (%BLUP)	h^2^
GBLUP	0.813	33.6	0.268
LASSO	0.849	39.6	0.293
BA_4df	0.914	50.3	0.291
BA_edf	0.937	54.1	0.285
BBt_4df	0.938	54.3	0.287
BBt_edf	0.935	53.8	0.287
BBn	0.94	54.6	0.286

Previous studies reported in the literature, have shown a superiority of Bayes B method over Bayes A (e.g. [1]). The results obtained here showed that the superior performance of Bayes B over Bayes A disappears when the parameters of the prior distribution of the SNP effect are estimated from the data. In this study, the shape parameter df, for BB_edf and BA_edf were estimated to be around 1 for both methods (see Additional file 2). Fixing df to be 4, resulted in a slight reduction on the GEBV accuracy for Bayes A, but it has little impact on the accuracy of Bayes B. This would explain why previous studies have reported a better performance of Bayes B over Bayes A. An interesting result was on the estimation of the parameter π. The estimated value obtained with both BBn and BBt_4df was around 0.01, but for BBt this estimate was around 0.4. Surprisingly, this large difference in π had very small impact on the overall accuracy of the GEBV, suggesting the need of further study to refine the meaning of the π and its relationship with the true proportion of SNP, or QTL affecting the trait.

### QTL mapping and association analysis

The dataset simulated includes 5 simple additive QTLs (one on chromosome 1, 2 on chromosome 2 and 2 on chromosome 3), one imprinted QTL (chromosome 4) and 2 QTLs with epistasis effect (on chromosome 5).

**Figure 2:**
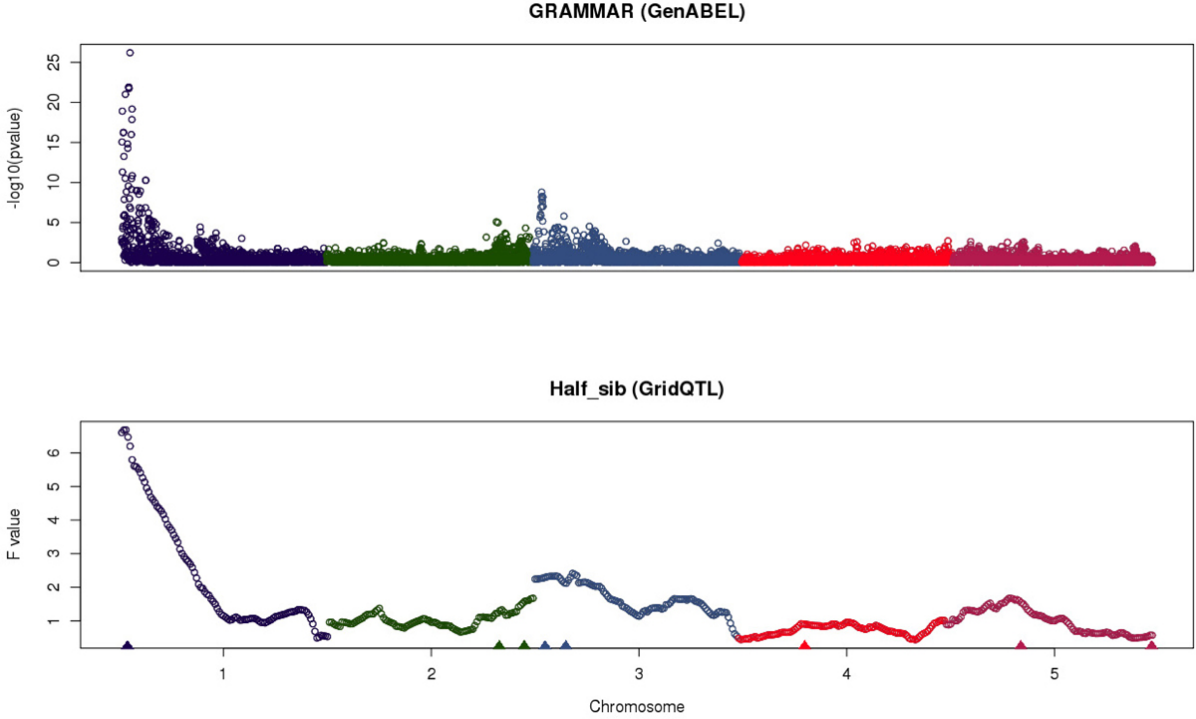
**Mapping profile for the linkage (Half-sib) and association analysis (position of simulated QTL were shown in triangles)**.

**Figure 3:**
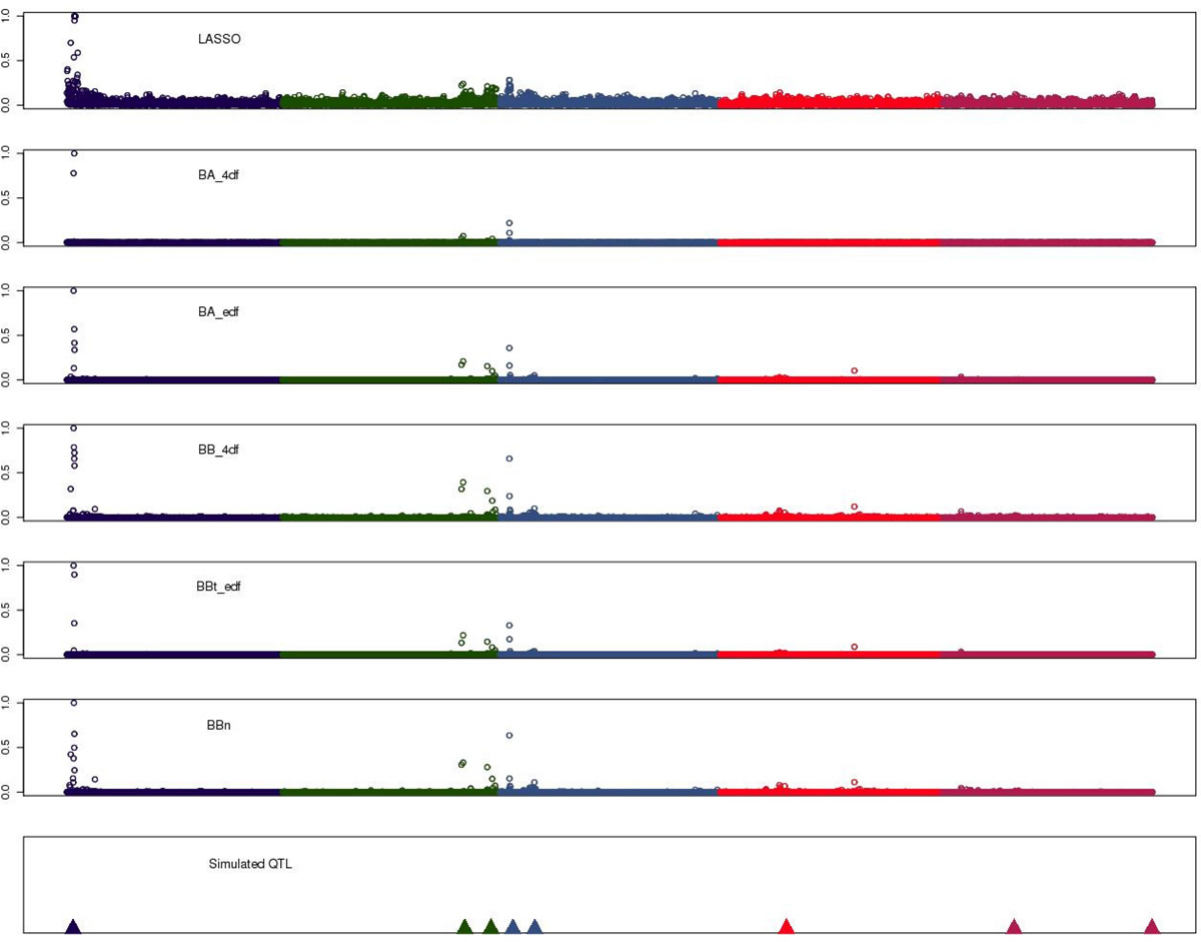
**Estimated SNP effect for the different genomic selection methods**.

Figure 2 shows interval mapping profiles for Half-sib QTL mapping and the association analysis and Figure 3 the absolute SNP effects (expressed as proportion to the SNP with largest effects) estimated with the different genomic selection methods. The QTL mapping methods were successful in identifying the major QTL on chromosome 1 and with some extend the other additive QTL (Figure 2). The different genomic selection methods also tended to assign greater effect to SNPs located in regions where the additive QTL were located (Figure 3). Within a QTL region (e.g. chr 1) all methods explained the QTL effect by selecting several linked SNP and assigning them a non zero effect. LASSO tended to select more SNPs with effect (Figure 3) than the Bayes A and Bayes B methods, but with smaller absolute effect (results not shown). BBt_edf and BA_edf required the lowest number of linked SNP to explain the QTL effect.

## Conclusions

Good consistency was observed for the results of all applied methods. For this specific dataset, the accuracy of Bayes A and B type of methods were better than those of GBLUP and LASSO. When all parameters of the prior distribution were estimated with the data, the results of Bayes A and Bayes B were the same. Fixing the shape parameter with Bayes A have a slight decrease in the accuracy but little effect on Bayes B. These results are consistent with previous studies reporting a superiority of Bayes B over Bayes A when the parameters of the prior distribution are assumed known.

## Competing interests

The authors declare that they have no competing interests.

## Authors' contributions

JN carried out the analyses and drafted the manuscript; RPW implemented the methods, all authors contributed to the design of the study and the final text of the manuscript.

## Supplementary Material

Additional file 1 **Correlation between GEBV estimated by different methods**. Click here for file

Additional file 2 **Posterior distribution of the shape parameter, df, for BA_edf and BBt_edf**. Click here for file
