# Supplementary figures and images for: Effect of the prior distribution of SNP effects on the estimation of total breeding value
Source: BMC Proc. 2012 May 21;6(Suppl 2):S6. doi: 10.1186/1753-6561-6-S2-S6 (PMC3363160; doi:10.1186/1753-6561-6-S2-S6)

**GBLUP**

**LASSO**

**BA\_edf**

**BA\_4df**

**BBt\_edf**

**BBt\_4df**

**BBn**

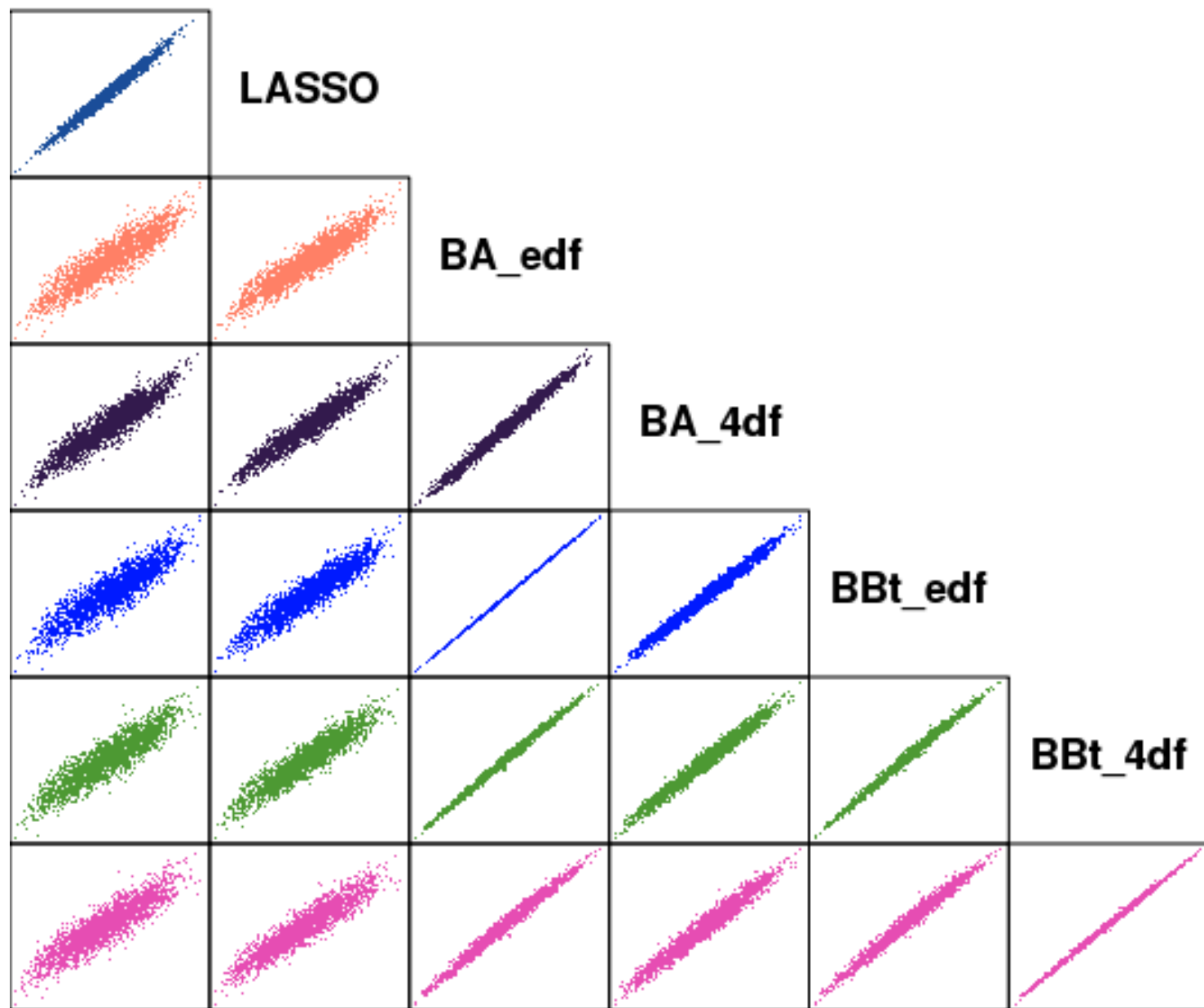

Supplement: Additional file 1 — Correlation between GEBV estimated by different methods. [file 1753-6561-6-S2-S6-S1.pdf]

**BBt\_edf**

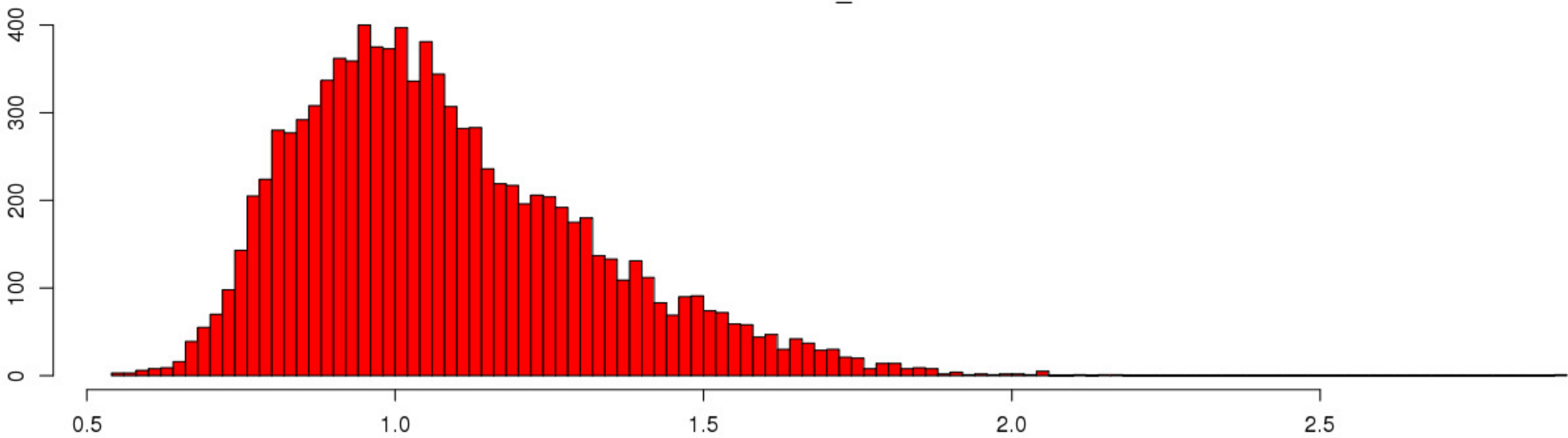

**BA\_edf**

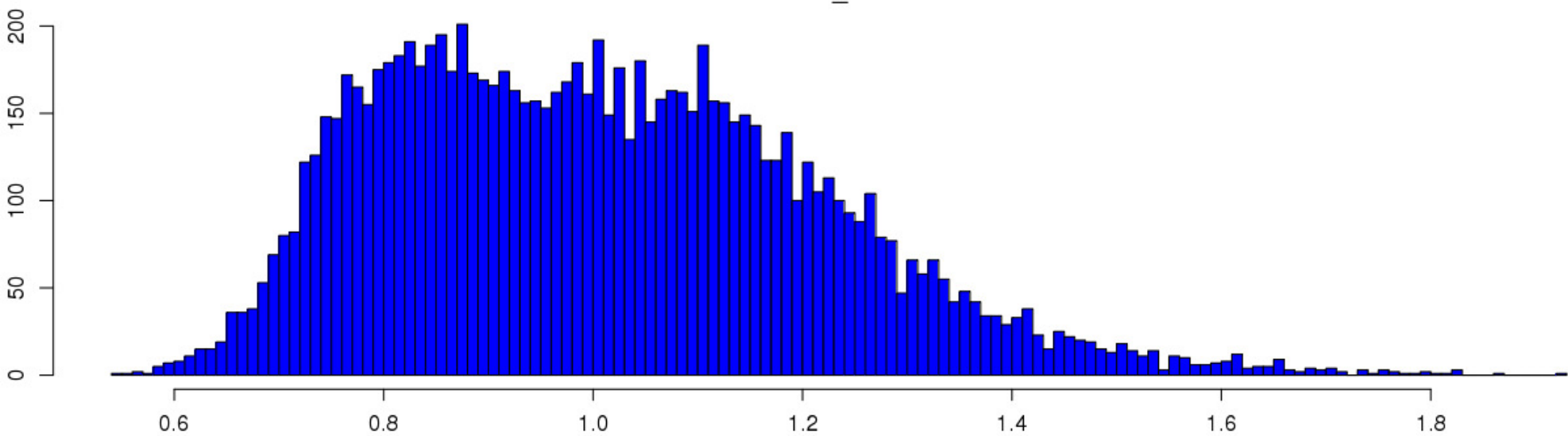

Supplement: Additional file 2 — Posterior distribution of the shape parameter, df, for BA_edf and BBt_edf. [file 1753-6561-6-S2-S6-S2.pdf]
